# Pre-Column Derivatization HPLC Procedure for the Quantitation of Aluminium Chlorohydrate in Antiperspirant Creams Using Quercetin as Chromogenic Reagent

**DOI:** 10.1007/s10337-014-2722-9

**Published:** 2014-07-10

**Authors:** Eleni Kalogria, Athanasia Varvaresou, Spyridon Papageorgiou, Evaggelia Protopapa, Ioannis Tsaknis, Alexios Matikas, Irene Panderi

**Affiliations:** 1 Division of Pharmaceutical Chemistry, Department of Pharmacy, University of Athens, Panepistimiopolis, Zografou, 157 71 Athens, Greece; 2 Department of Aesthetics and Cosmetology, The School of Health and Caring Professions, Technological Education Institution of Athens, Ag. Spyridonos str., 122 10 Athens, Greece; 3 Medical Oncology Department, University Hospital of Heraklion, Mainz, Greece

**Keywords:** RP-HPLC, Pre-column derivatization, Aluminium chlorohydrate, Quercetin, Method development, Validation

## Abstract

This article describes the development and validation of a selective high-performance liquid chromatography method that allows, after liquid–liquid extraction and pre-column derivatization reaction with quercetin, the quantification of aluminium chlorohydrate in antiperspirant creams. Chromatographic separation was achieved on an XTerra MS C18 analytical column (150 × 3.0 mm i.d., particle size 5 μm) using a mobile phase of acetonitrile:water (15:85, v/v) containing 0.08 % trifluoroacetic acid at a flow rate of 0.30 mL min^−1^. Ultraviolet spectrophotometric detection at 415 nm was used. The assay was linear over a concentration range of 3.7–30.6 μg mL^−1^ for aluminium with a limit of quantitation of 3.74 μg mL^−1^. Quality control samples (4.4, 17.1 and 30.6 μg mL^−1^) in five replicates from five different runs of analysis demonstrated intra-assay precision (% coefficient of variation <3.8 %), inter-assay precision (% coefficient of variation <5.4 %) and an overall accuracy (% recovery) between 96 and 101 %. The method was used to quantify aluminium in antiperspirant creams containing 11.0, 13.0 and 16.0 % (w/w) aluminium chlorohydrate, respectively.

## Introduction

Aluminium salts, such as aluminium chlorohydrate that has been introduced in the market since 1947, are the active ingredients of antiperspirant in underarm and bodycare cosmetics applied to the underarm and breast area [1]. The effects of widespread, long-term and increasing use of aluminium salts in these cosmetics remain unknown. Aluminium is known to have a genotoxic profile, capable of causing both DNA alterations and epigenetic effects, and this could be consistent with a potential role in breast cancer [2]. Results reported in a recent research demonstrate that aluminium in the form of aluminium chloride or aluminium chlorohydrate can interfere with the function of oestrogen receptors of MCF7 human breast cancer cells, both in terms of ligand binding and in terms of oestrogen-regulated reporter gene expression [3]. This adds aluminium to the increasing list of metals capable of interfering with oestrogen action and termed metalloestrogens [4]. The use of aluminium antiperspirants has also been linked with increased risk of Alzheimer’s disease due to the possible systemic accumulation of aluminium; however this hypothesis remains controversial [5–7].

Although much research has been undertaken into the antiperspirant properties of a number of aluminium salts, very little of this work is focused upon the quantification of these products in cosmetic formulations. Given that the toxicity of aluminium has been widely recognized, reducing the concentration of this metal in antiperspirants is a matter of urgency and there is a real need to set up analytical methods to quantitate aluminium salts in underarm cosmetics.

Hyphenated techniques by combining various chromatographic techniques with atomic spectrometry/mass spectrometry are the most efficient for the determination of aluminium in human body fluids [8, 9]. Recently, high-performance liquid chromatography interfaced to flame atomic spectrometry (HPLC–FAAS) [10] and a post-column derivatization procedure with 4,5-dihydroxy-1,3 benzene disulfonic acid disodium salt [11] have been used to quantify aluminium fluoride complexes in groundwater samples. Several high-performance liquid chromatography methods have been reported for the analysis of aluminium in various matrixes (aqueous, serum and wine samples), the majority of which include pre-column derivatization with lumogallion [12–14], morin [15], quercetin [16], *N*-*o*-vanillidine-2-amino-p-cresol [17] and 8-hydroxyquinoline [18, 19]. Flow and sequential injection methods have been reported for the spectrofluorimetric determination of aluminium in pharmaceutical formulations using chromotropic acid as chromogenic reagent [20]. Recently, the binding sites of quercetin with the Al^3+^ ion have been identified using solid-state NMR [21].

To the best of our knowledge, no methodology has been described in the literature to quantify aluminium salts in cosmetics. Thus, the principal aim of this work was to optimize and validate an analytical procedure for the quantitative determination of aluminium chlorohydrate in antiperspirant cream samples based on a pre-column derivatization procedure using quercetin, 2-(3,4-dihydroxyphenyl)-3,5,7-trihydroxychromen-4-one, as the derivatization reagent. The method is the first reported application and could be used for routine analysis of antiperspirant creams containing aluminium chlorohydrate, as it complies well with the validation requirements in the cosmetic industries [22].

## Experimental

### Materials and Reagents

All solvents were of HPLC grade and were purchased from Merck (Darmstadt, Germany). Trifluoroacetic acid and ammonium acetate of analytical reagent grade were obtained from Merck (Darmstadt, Germany). Water was deionized and further purified by means of a Milli-Q Plus Water Purification System, Millipore Ltd. Aluminium chlorohydrate solution 50 % (w/w) and quercetin were purchased from Sigma-Aldrich (Steinheim, Germany).

Cosmetic antiperspirant creams containing 11, 13 and 16 % (w/w) aluminium chlorohydrate were produced in the Department of Aesthetics and Cosmetology of the Technological Educational Institution of Athens, Greece. The excipients present in creams were: distarch phosphate, allantoin, ceteareth-12, ceteareth-20, glyceryl stearate, cetyl alcohol, octyl stearate, dimethicone, triethyl citrate, methyl paraben, ethyl paraben, propyl paraben, cyclomethicone, PPG-25-laureth-25, parfum and aqua.

### Instrumentation

The chromatographic equipment used consisted of a Spectra Series P100 isocratic pump (SP ThermoSeparation, UK) and a Rheodyne Model 7725i injector (Rheodyne California, CA, USA) with a 20 μL loop. The detection was performed using a Waters 486 UV–Vis detector (Waters, Milford, MA, USA) operated at 415 nm. Data acquisition and analysis were performed using Empower software (Waters, Milford, MA, USA).

All glassware containers were carefully treated with 2.0 M nitric acid for more than 48 h and rinsed with water.

### Liquid Chromatographic Conditions

Chromatography was performed at ambient conditions on an XTerraMS C18 reversed HPLC analytical column (150.0 × 3.0 mm i.d., 5 μm particle size), Waters (Milford, MA, USA). The mobile phase consisted of acetonitrile: water (15:85, v/v) containing 0.08 % trifluoroacetic acid. It was filtered through a 0.45 μm nylon-membrane filter, GelmanSciences (Northampton, UK), and degassed under vacuum prior to use. A flow rate of 0.30 mL min^−1^ with a column inlet pressure of 1,450 psi was used to separate the excess of quercetin from the aluminium–quercetin complex. Chromatography was performed at 25 ± 2 °C with a chromatographic run time of <7.0 min.

### Stock and Working Standard Solutions

Stock standard solution of aluminium chlorohydrate, containing 875.0 μg mL^−1^ aluminium, was prepared by appropriate dilution of the aluminium chlorohydrate solution 50 % (w/w) in water. Stock standard solution of the reagent, quercetin, 500.0 μg mL^−1^, was prepared by dissolving the appropriate amount of the compound in methanol. These solutions were stored in the dark under refrigeration and were found to be stable for a period of 4 weeks.

A working standard solution of aluminium chlorohydrate, containing 43.75 μg mL^−1^ of aluminium, was prepared by subsequent dilution of the above-mentioned stock standard solution in water. The working standard solution was freshly prepared every week and stored in the dark at 4 °C.

### Calibration Spiked Cream Samples and Quality Control Sample Preparation

Calibration spiked cream samples were freshly prepared every working day at the concentration levels of 3.7, 4.4, 6.6, 10.9, 15.3, 17.1, 19.7, 26.2 and 30.6 μg mL^−1^ for aluminium by addition of the appropriate aliquot of the above-mentioned working standard solution of the analyte to 25 mg of placebo cream samples. Quality control (QC) samples were prepared independently, in an analogous manner as the calibration spiked cream samples, using separate stock standard solution of the analyte. QC samples were prepared in placebo cream at three concentration levels (4.4, 17.1 and 30.6 μg mL^−1^) for aluminium.

### Sample Preparation and Derivatization Procedure

Extraction, cleanup and derivatization procedures for cream samples were carried out according to the following steps. Exactly, 25 mg of cream sample was transferred into a 20 mL volumetric flask with 2 mL of acetonitrile and diluted to volume with HCl 0.01 M. The mixture is shaken for 2 min and a 2 mL portion of this solution is centrifuged at 18,000×*g* for 20 min. In 1 mL of the aqueous phase (bottom layer) 500 μL of* tert*-butyl methyl ether is added and the mixture is vigorously mixed on a vortex mixer for 2 min and centrifuged at 18,000×*g* for 15 min. The organic layer is discarded and 100 μL aliquot of this solution is treated with 500 μL of 1.0 M ammonium acetate–acetic acid buffer (pH 4.5) and 1.0 mL of a 500.0 μg mL^−1^ quercetin solution. The solution is vigorously mixed on a vortex mixer for 1 min and diluted to 10.0 mL with a mixture of acetonitrile–methanol (10:90, v/v). A 20 μL aliquot is then injected into the chromatographic system.

### Validation Procedure

To evaluate the linearity of the proposed method, the calibration spiked cream samples were prepared and analysed in duplicate on three different analytical runs. Quantitation was performed using the peak area of aluminium–quercetin complex. QC samples were processed in five replicates at each concentration (4.4, 17.1 and 30.6 μg mL^−1^) for five different analytical runs to evaluate the intra- and inter-assay accuracy and precision.

The standard addition method was used to evaluate the effect of the excipients on the determination of aluminium chlorohydrate. Thus, six equal amounts of cream samples equivalent to 0.350 mg of aluminium chlorohydrate (0.044 mg of aluminium) were spiked with different amounts of the working standard solution of the analyte. The spiked cream samples were then analysed as mentioned in the assay procedure.

## Results and Discussion

### Optimization of the Pre-Column Reaction Procedure

**Fig. 1:**
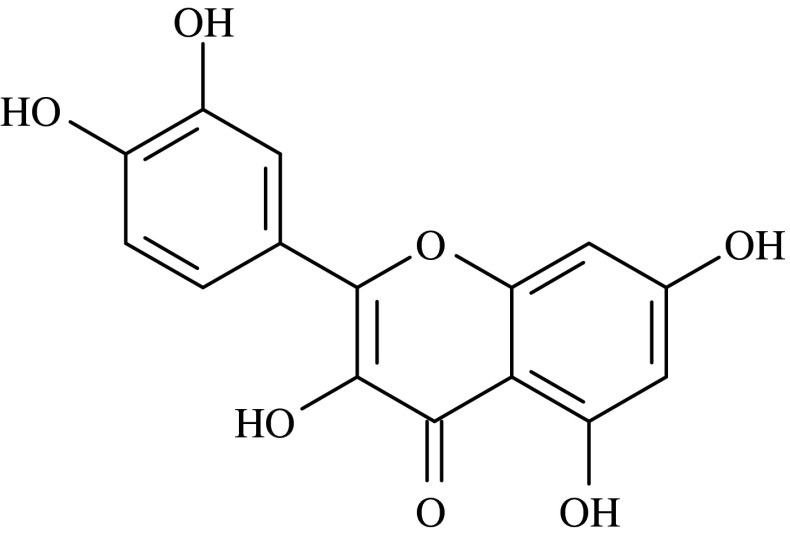
Chemical structure of quercetin

Quercetin (Fig. 1) possesses two possible chelating sites, 3-hydroxy-4-oxo and 5-hydroxy-4-oxo systems. Literature survey reveals that aluminium is bonded to 3-hydroxy-4-oxo system and forms predominantly a 1:1 complex with quercetin. There is no evidence that 3,4 dihydroxyflavone forms a complex in acid solution [16, 23]. The applicability of quercetin as chromogenic reagent for the analysis of aluminium in antiperspirant creams containing aluminium chlorohydrate was thoroughly investigated. In every step of the optimization procedure all the contributing factors but one remain constant and the optimized value is used for the next experiment.

**Fig. 2:**
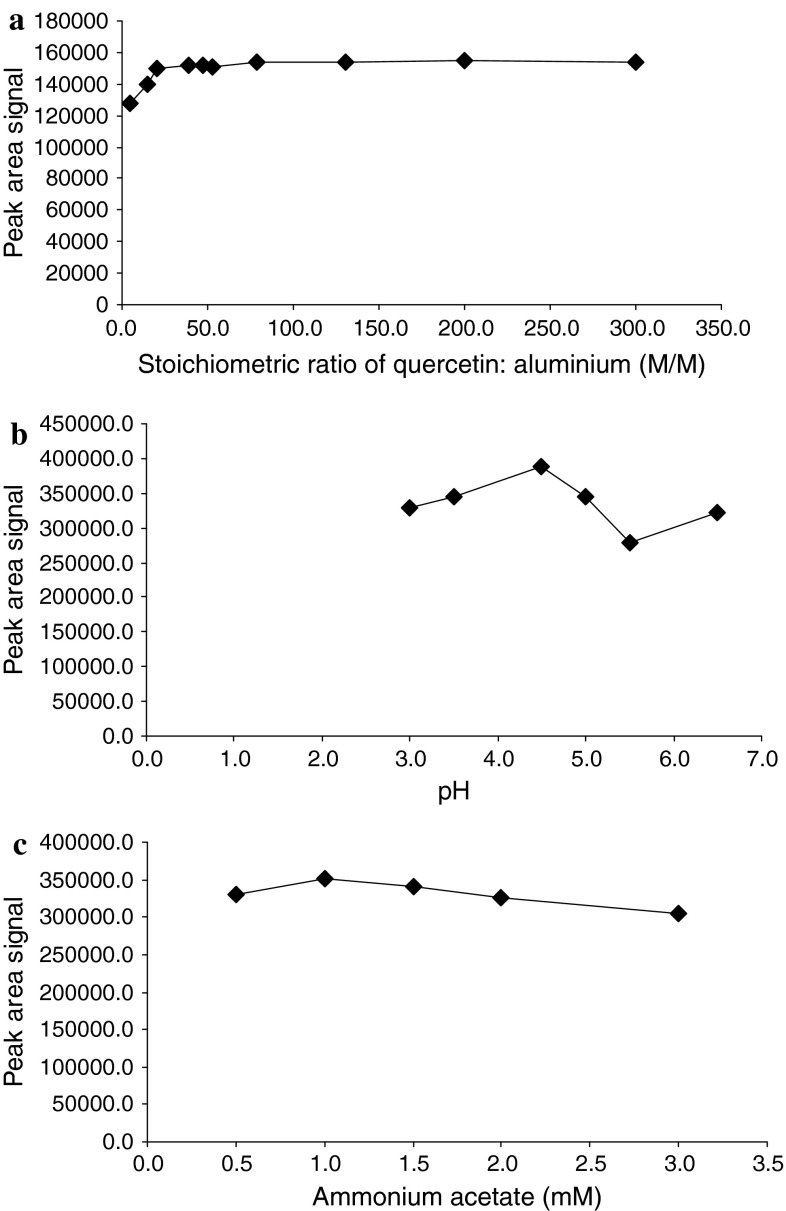
Optimization of the derivatization reaction. **a** Plots of the signal of the quercetin–aluminium complex versus the stoichiometric ratio of quercetin:aluminium, M/M, **b** plots of the signal of the quercetin–aluminium complex versus the concentration of ammonium acetate (mM) in the reaction medium and **c** plots of the signal of the signal of the aluminium–quercetin complex versus the pH value of the reaction medium

The effect of the stoichiometric ratio of quercetin:aluminium, M/M on the peak area signal of quercetin–aluminium complex was examined over the range of 5:1–300:1 M/M. The results indicated that once the molar proportion of quercetin:aluminium exceeds 20:1 M/M, the peak area of the complex does not significantly increase, remains constant up to 200:1 (Fig. 2a), and covers the range of the calibration curve.

The effects of the concentration (0.5–3.0 M) and pH (3.0–6.5) of the ammonium acetate–acetic acid buffer solution that was used as the reaction solvent were also investigated. The complex formation reached the maximum reaction yield in 1.0 M ammonium acetate–acetic acid buffer solution, whereas further increases in the concentration of ammonium acetate decreased the peak area of the complex (Fig. 2b). Also, the pH of the buffer solution is a critical factor for the complex formation. The maximum reaction yield was achieved at pH 4.5, whereas at pH values greater than 5.5 the signal decreased rapidly (Fig. 2c). This effect can be attributed to the fact that under acidic aqueous solutions, the aluminium ion exists mainly as Al^3+^ and an increase in pH results in the formation of complexes of aluminium with hydroxide and finally the formation of insoluble aluminium hydroxide at neutral pH. The optimum conditions were the following: ratio of quercetin:aluminium greater than 20:1, M/M in 1.0 M ammonium acetate–acetic acid buffer solution pH 4.5. The complex was found to be stable for 80 min prior to the injection into the HPLC system, which is adequate time for the chromatography.

### Optimization of Chromatographic Conditions

Chromatography was performed using an XTerra MS C18 (150.0 × 3.0 mm i.d., 5 μm particle size) column, while chromatographic conditions were optimized to separate quercetin–aluminium complex from the excess of quercetin and the cream matrix excipients. Methanol and acetonitrile were tried as organic modifiers in the mobile phase in combination with water. In the present study, acetonitrile was preferred to methanol as it gave better peak shape. It was found that an increase in the content of acetonitrile as organic modifier in the mobile phase could improve peak shape, whereas an increase in water content broadened the peak. An increase in the retention of the complex is observed with increasing trifluoroacetic acid from 0.02 to 0.12 % (v/v) and improved peak shape. Thus, a mobile phase consisting of 15 % acetonitrile in water containing 0.08 % trifluoroacetic acid was used as the optimum. Each chromatographic run was completed within 7.0 min.

**Fig. 3:**
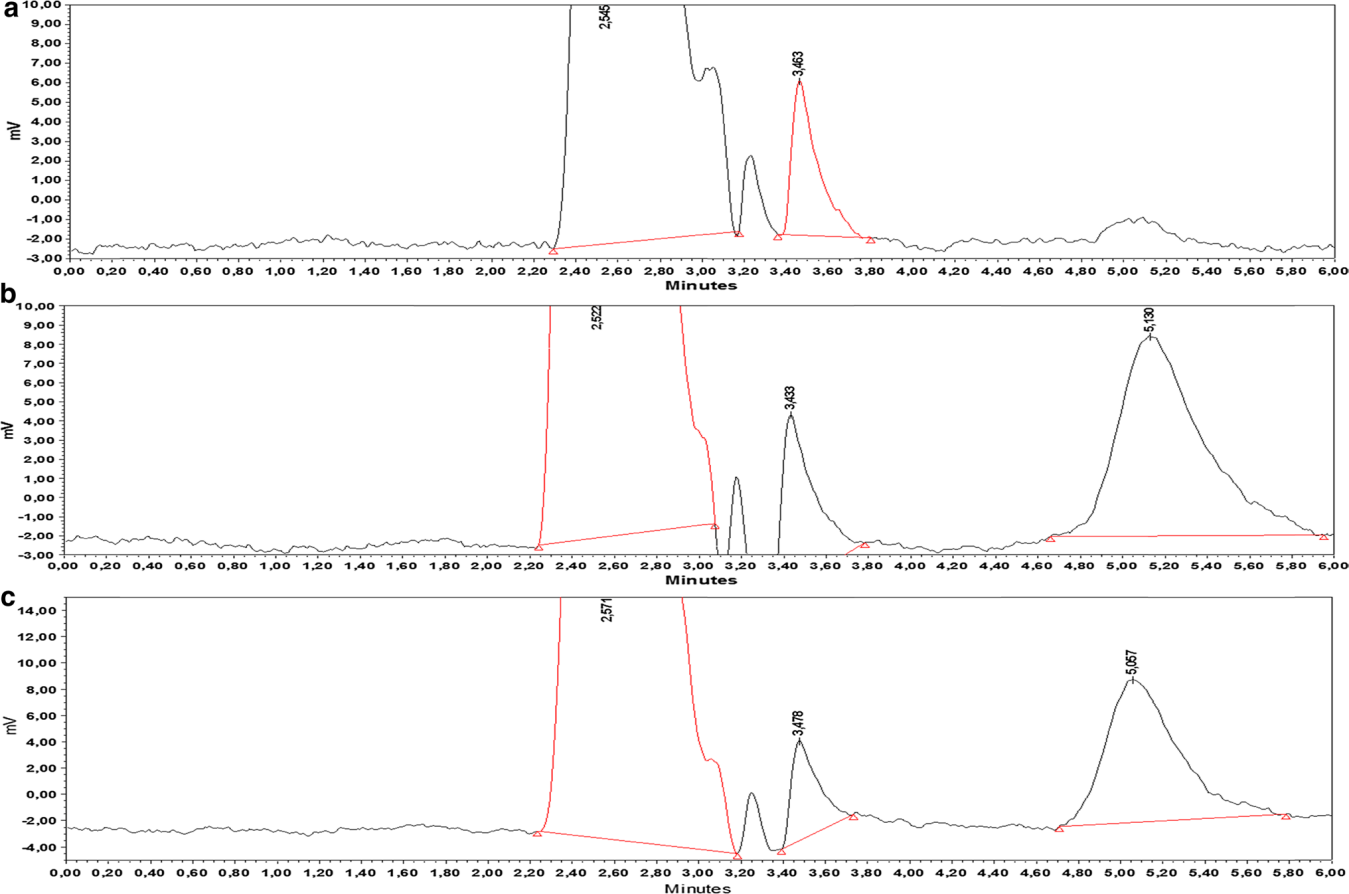
**a** Representative RP-HPLC chromatograms obtained from the analysis of** a** a blank cream matrix sample. **b** A calibration spiked cream sample containing 19.7 μg mL^−1^ aluminium and **c** a cream sample containing 17.0 μg mL^−1^ aluminium. Chromatographic conditions: RP-HPLC on an XTerraMS C18 analytical column; mobile phase: acetonitrile:water (15:85, v/v) containing 0.08 % trifluoroacetic acid; flow rate 0.30 mL min^−1^ and a UV detector at 415 nm

The selectivity of the proposed chromatographic procedure is illustrated in Fig. 3 with a representative HPLC chromatogram obtained from the analysis of a placebo cream sample without the addition of aluminium chlorohydrate (Fig. 3a) along with a calibration spiked cream sample containing 19.7 μg mL^−1^ of aluminium (Fig. 3b) and a chromatogram obtained from the analysis of a cream sample containing 17.00 μg mL^−1^ aluminium (Fig. 3c). Under the current chromatographic conditions, complete separation among the aluminium–quercetin complex and the excipients is achieved and the complex is eluted at 6.07 min.

### Statistical Analysis of Data

Calibration spiked cream samples of aluminium chlorohydrate were analysed in triplicate in three analytical runs for the calibration procedure. Linear relationships between the peak area signals of aluminium–quercetin complex and the corresponding concentrations of aluminium were observed as shown by the results presented in Table 1; the correlation coefficient was greater than 0.997. Back-calculated concentrations in the calibration curves were <3.4 % of the nominal, which are in agreement with international guidelines. The insignificance of intercepts that was proven by a Student’s *t* test indicates that there is no effect from the cream’s excipients.

**Table 1:** Analytical concentration parameters of the calibration equations for the determination of aluminium, by pre-column derivatization HPLC method

Medium	Concentration range (μg mL^−1^)	Regression equation^a^	*r* ^b^	Standard deviation slope intercept		*S*_r_^c^	*α*/*S*_α_^d^
Water samples							
Run1	3.7–30.6	*S*_Al_ = 10301 × *C*_Al_ + 2466	0.998	177	3,152	2,126	1.27
Spiked cream samples							
Run 1	3.7–30.6	*S*_Al_ = 9750 × *C*_Al_ + 2092	0.998	198	3,651	2,780	0.57
Run 2	3.7–30.6	*S*_Al_ = 9762 × *C*_Al_ + 68	0.998	119	2,203	2,884	0.03
Run 3	3.7–30.6	*S*_Al_ = 9756 × *C*_Al_ − 1659	0.997	119	2,188	2,865	0.76
Mean of three calibration curves over a period of 1 month							
Spiked cream samples	3.7–30.6	*S*_Al_ = 9756 × C_Al_ + 167	>0.997	6.1	1,877	<2,780	<0.76

^a^Peak area signal of aluminium, *S*_Al_, versus the corresponding concentration of aluminium, *C*_Al_

^b^Correlation coefficient

^c^Standard error of the estimate

^d^Theoretical value of *t* at *P* = 0.05 and *f* = *n* − 2 = 6 df, 2.45

The limit of detection, LOD, and the limit of quantification, LOQ, for aluminium were determined according to the definitions of ICH Topic Q2B [22]. In particular, the LOD was calculated using the equation LOD = 3.3 × *S*a/*b*, and it was found to be at the level of 1.24 μg mL^−1^ while the limit of quantification, LOQ, was attained using the equation LOQ = 10 × *S*a/*b* (where *b* is the slope and *S*a is the standard deviation of the intercept, a, of the regression line) and it was found to be at the level of 3.74 μg mL^−1^.

One-way analysis of variance was used to evaluate the intra- and inter-assay precision. Results presented in Table 2 indicate that intra-assay relative standard deviation values, %RSD, were between 0.8 and 3.7 % for the analyte, while the inter-assay %RSD was no more than 5.5 %. The overall assay was assessed by % recovery which ranged from 96 to 101 %.

**Table 2:** Accuracy and precision evaluation of quality control samples for aluminium (*n* = 5 runs, five replicates per run)

Compound	Concentration (μg mL^−1^)		
Aluminium added concentration	4.4	17.1	30.6
Run 1 (mean ± SD)	4.18 ± 0.15	17.06 ± 0.11	30.28 ± 0.31
Run 2 (mean ± SD)	4.08 ± 0.25	17.41 ± 0.23	30.38 ± 0.25
Run 3 (mean ± SD)	4.22 ± 0.08	17.32 ± 0.13	30.36 ± 0.29
Run 4 (mean ± SD)	4.24 ± 0.13	17.39 ± 0.11	30.48 ± 0.19
Run 5 (mean ± SD)	4.38 ± 0.11	17.34 ± 0.17	30.51 ± 0.23
Overall mean	4.22	17.31	30.40
Intra-assay RSD (%)^a^	3.7	0.9	0.8
Inter-assay RSD (%)^a^	5.5	1.8	0.5
% Recovery^b^	96	101	99

^a^Coefficient of variation; intra- and inter-assay RSDs were calculated by ANOVA

^b^% Recovery = [(overall mean assayed concentration × 100)/(added concentration)]

A number of organic solvents such as hexane, diethyl ether and ethyl acetate were tested for the liquid extraction procedure and led to poor recoveries.* Tert*-butyl methyl ether was finally chosen as the optimum extraction solvent. The recovery of the extraction procedure was evaluated by comparing the slope of the regression equation obtained from the analysis of calibration spiked cream samples over the slope of the regression equation obtained from the analysis of calibration samples prepared in water solution and analysed immediately without sample preparation procedure (Table 1). The data, under the optimum extraction conditions, indicate a recovery of 95 % for aluminium.

To verify the robustness of the method, small deliberate variations were introduced around the optimal conditions and the influence of these variations in the retention time, capacity factor, tailing factor and concentration of aluminium in cream samples was thoroughly investigated. The parameters selected to examine were the percentages of acetonitrile and trifluoroacetic acid in the mobile phase and the wavelength of UV detection. Replicate injections (*n* = 3) of a cream sample containing 11 % w/w aluminium chlorohydrate and processed according to the sample preparation procedure were performed under small changes of the aforementioned parameters. The evaluation of the method robustness (Tables 3, 4) indicates that there is no significant difference in the measured responses after small variations of the selected parameters.

**Table 3:** Robustness evaluation of the pre-column derivatization HPLC method for the determination of aluminium chlorohydrate in antiperspirant creams

Chromatographic changes	Measured responses			
Parameters^a^	*t*_r_^b^	*k*′^c^	*T* ^d^	Concentration of aluminium chlorohydrate % (w/w)
A wavelength of UV detection (414–420 nm)				
Mean (%RSD)	5.07 (0.4)	1.31 (0.8)	1.23 (0.3)	11.3 (2.5)
B % trifluoroacetic acid in the mobile phase (0.75–0.85 % v/v)				
Mean (%RSD)	5.14 (0.9)	1.29 (1.7)	1.28(0.5)	11.4 (3.3)
C % of acetonitrile in the mobile phase (69–71 % v/v)				
Mean (%RSD)	5.12 (2.1)	1.32 (3.4)	1.24 (0.7)	11.5 (3.5)

^a^Three parameters (A, B and C) were slightly changed at three levels (1, 0, −1); each time a parameter was changed from level (0), the others remained at level (0)

^b^retention time

^c^capacity factor

^d^tailing factor

**Table 4:** Quantification of aluminium chlorohydrate in antiperspirant creams by a pre-column derivatization HPLC method

Lot no.	% Label claim in aluminium chlorohydrate	Experimental aluminium chlorohydrate concentration/100 mg cream mean value ± SD (*n*=10)	% Recovery	% *E*_r_
1	11	10.6 ± 0.6	96 ± 5	−3.2
2	13	12.7 ± 0.5	97 ± 4	−2.6
3	16	15.4 ± 0.6	96 ± 4	−3.9

### Application of the Method to the Analysis of Real Samples

The proposed method was evaluated in the assay of three different lots of antiperspirant creams containing 11, 13 and 16 % (w/w) of aluminium chlorohydrate, and the percent label claims for aluminium chlorohydrate were found to be 97 ± 5, 97 ± 4 and 96 ± 4, respectively.

To further assess the specificity of the proposed method, recovery studies were also performed by spiking cream samples with known and different amounts of aluminium chlorohydrate. The regression line of the instrumental response versus the added concentration of aluminium is plotted and the negative intercept on the concentration axis (*x*-axis) corresponds to the concentration of the analyte in the cream sample. This value is given by the ratio of the intercept and the slope of the regression line [24], which were found to be 36,890 ± 7,817 and 17,853 ± 423, respectively. The label claim for aluminium chlorohydrate using the standard addition method was found to be 10.4 % w/w.

## Conclusions

Aluminium chlorohydrate is the active ingredient of antiperspirant in underarm and bodycare cosmetics applied to the underarm and breast area. No methodology has been previously described to quantitate aluminium chlorohydrate in antiperspirant creams. The proposed pre-column derivatization HPLC method using quercetin as chromogenic reagent was evaluated over the linearity, precision, accuracy and specificity and proved to be convenient and effective for the determination of aluminium chlorohydrate in creams.

## References

[1] Laden K (1988). Felger CB Antiperspirants and Deodorants: Cosmetic Science and Technology Series.

[2] Darbre PD, Mannello F, Exley C (2013). J Inorg Biochem.

[3] Scheel C, Weinberg RA (2012). Semin Cancer Biol.

[4] Darbre PD (2006). J Appl Toxicol.

[5] Exley C (1998). Mol Med Today.

[6] Pohanka M (2014). Environ Toxicol Pharmacol.

[7] Di Lorenzo F, Di Lorenzo B (2013). Neuro Endocrinol Lett.

[8] Chen B, Zeng Y, Hu B (2010). Talanta.

[9] Murko S, Milacic R, Kralj B, Scancar J (2009). Anal Chem.

[10] Frankowski M, Zioła-Frankowskaa A, Siepak J (2010). Talanta.

[11] Frankowski M (2012). Microchemical J.

[12] Wu J, Zhou CY, Chi H, Wong MK, Lee HK, Ong HY, Ong CN (1995). J Chromatogr B Biomed Appl.

[13] Ren JL, Zhang J, Luo JQ, Pei XK, Jiang ZX (2001). Analyst.

[14] Lee BL, Chua LH, Ong HY, Yang HG, Wu J, Ong CN (1996). Clin Chem.

[15] Lian HZ, Kang YF, Yasin A, Bi SP, Shao DL, Chen YJ, Dai LM, Tian LC (2003). J Chromatogr A.

[16] Lian H, Kang Y, Bi S, Askin Y, Shao D, Li D, Chen Y, Dai L, Gan N, Tian L (2004). Talanta.

[17] Kara D, Fisher A, Hill SJ (2008). Anal Chim Acta.

[18] Kelly MT, Blaise A (2006). J Chromatogr A.

[19] Kashimura K, Mizushima Y, Hoshino E, Matsubara S (2003). J Chromatogr B.

[20] Themelis DG, Kika FS (2006). J Pharm Biomed Anal.

[21] Ahmedova A, Paradowska K, Wawer I (2012). J Inorg Biochem.

[22] International Conference on Harmonisation (ICH) (1996) Tripartite guideline validation of analytical procedures: text and methodology Q2 (R1): current step 4 version parent guideline dated 27 October 1994, (Complementary Guideline on Methodology dated 6 November 1996 incorporated in November 2005)

[23] Cornard JP, Dangleterre L, Lapouge C (2005). J Phys Chem A.

[24] Miller JN (2005). Miller JC Statistics and Chemometrics for Analytical Chemistry.

